# Supplementary material for: Intraspecific Variation in Functional Traits of Medicago sativa Determine the Effect of Plant Diversity and Nitrogen Addition on Flowering Phenology in a One-Year Common Garden Experiment
Source: Plants (Basel). 2023 May 16;12(10):1994. doi: 10.3390/plants12101994 (PMC10223969; doi:10.3390/plants12101994)
Supplement: Supplementary file 1 [file plants-12-01994-s001.zip › plants-2248050-supplementary.pdf]

Supplementary Information

Figure S1 Design and photo of our experiments.

(a)

(b)

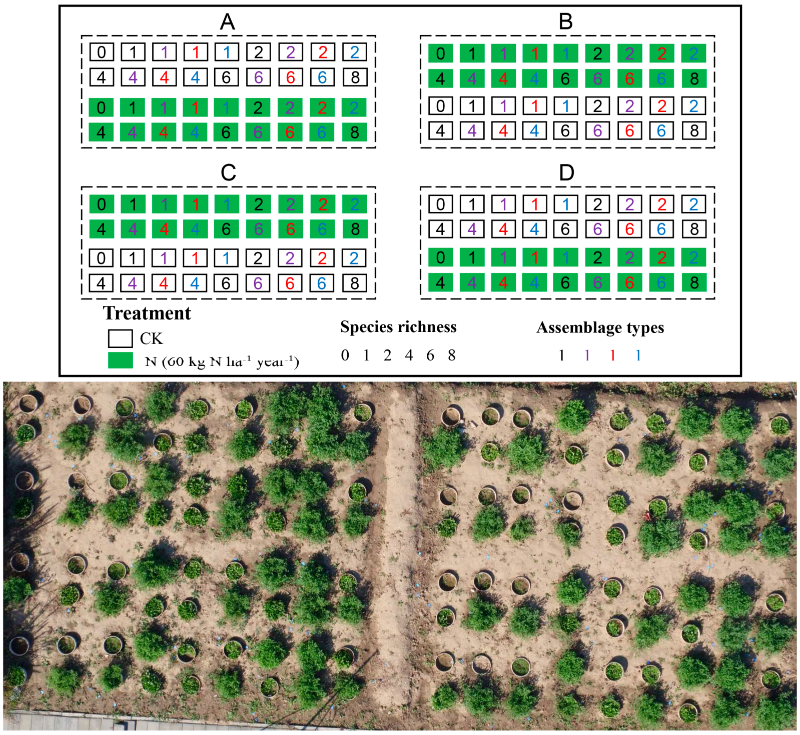

Shown are (a) experiment design, and (b) overall view of the experimental plots.

Figure S2 Photos of experimental arrangement.

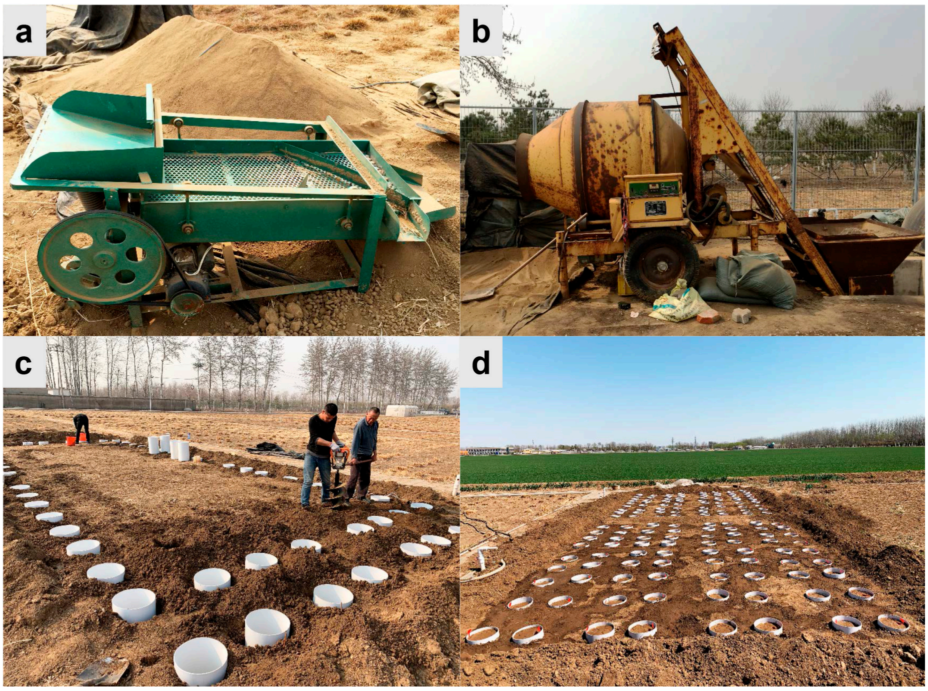

Shown are (a) sieving soil, (b) mixing soil and sand, (c) burying the pipes, and (d) overall view of the experimental plots.

Figure S3 The average biomass (a), relative biomass (b), relative abundance (c), and relative height (d) of *M. Sativa* along the plant species richness gradients under different nitrogen (N) addition levels. All the analyses were performed using the linear mixed effects models to test the effects of treatments (plant diversity and N addition) on plant traits of *M. Sativa*. Treatments were treated as fixed factors, block was treated as a random factor. Points and lines with different shape represent different N addition levels, points with different colour represent different plant diversity levels. N represents nitrogen addition, D represents plant diversity.

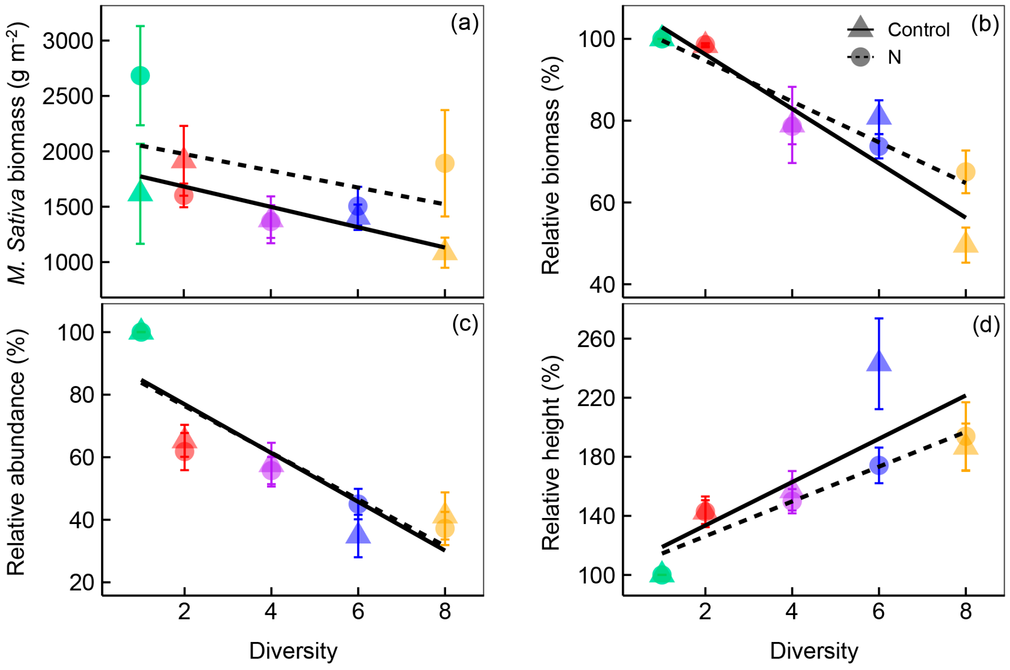

Figure S4 Pearson's correlation between the biotic and abiotic factors. The size and colour of the circles indicate the strength and sign of the correlation. N, nitrogen addition; D, plant diversity; FFD, first flowering day; LFD, last flowering day; FD, flowering duration; FN, flowering numbers; LM, leaf mass; LA, leaf area; LL, leaf length; LW, leaf width; SLA, specific leaf area; LN, leaf nitrogen content; LC, leaf carbon content; LCN, leaf carbon/ nitrogen ratio; AB, aboveground biomass; PA, plant abundance; PH, plant height; RB, relative biomass; RA, relative abundance; RH, relative height; AN, available soil nitrogen; SM, soil moisture; ST, soil temperature.

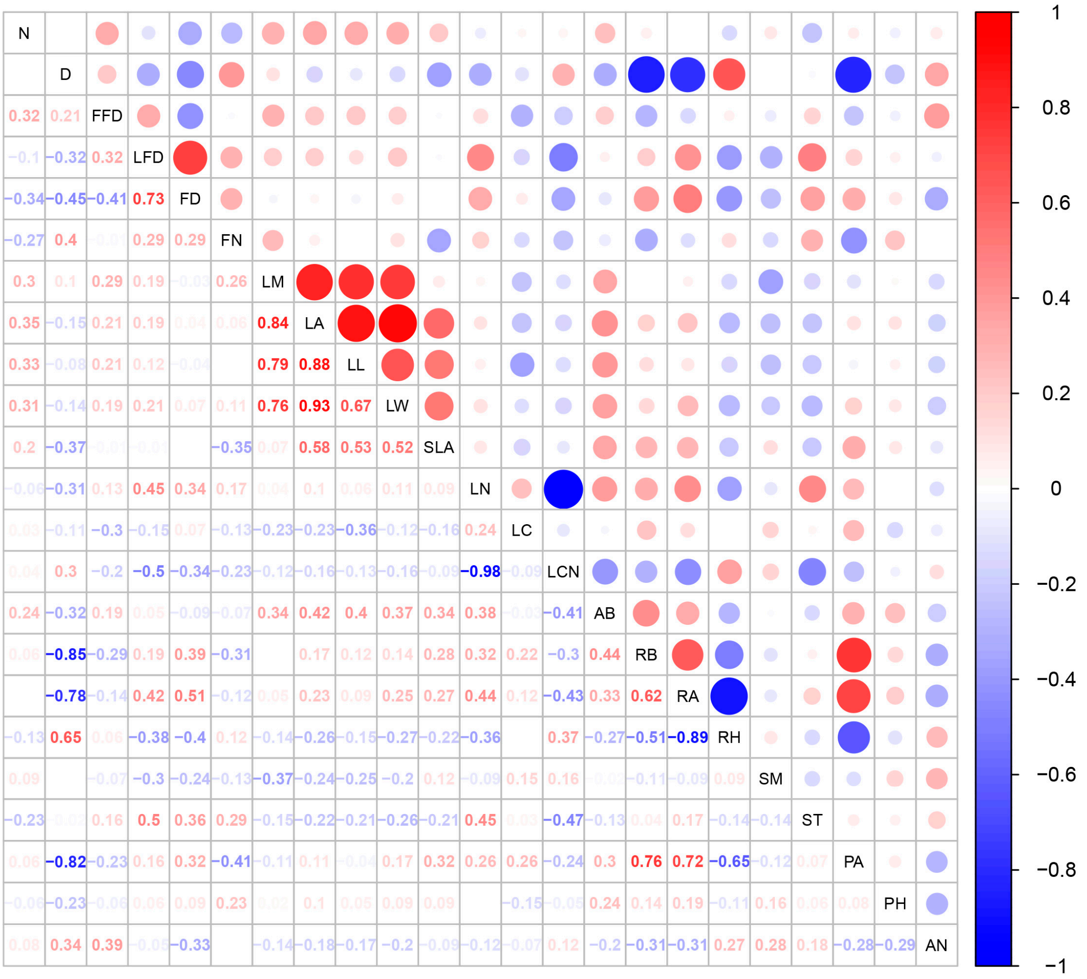

Figure S5 Relative contributions of light acquisition traits, nutrient acquisition traits, and abiotic factors to flowering events. Variation partitioning analysis was to identify the variance in the first flowering day (a), the last flowering day (b), flowering duration (c), and flowering numbers (d) explained by these three groups factors. Light acquisition traits including leaf mass, leaf area, leaf length, leaf width, specific leaf area, plant height, and relative height; nutrient acquisition traits including leaf nitrogen content, leaf carbon content, leaf carbon/ nitrogen ratio, aboveground biomass, plant abundance, plant height, relative biomass, and relative abundance; abiotic factors including available soil nitrogen, soil moisture, and soil temperature.

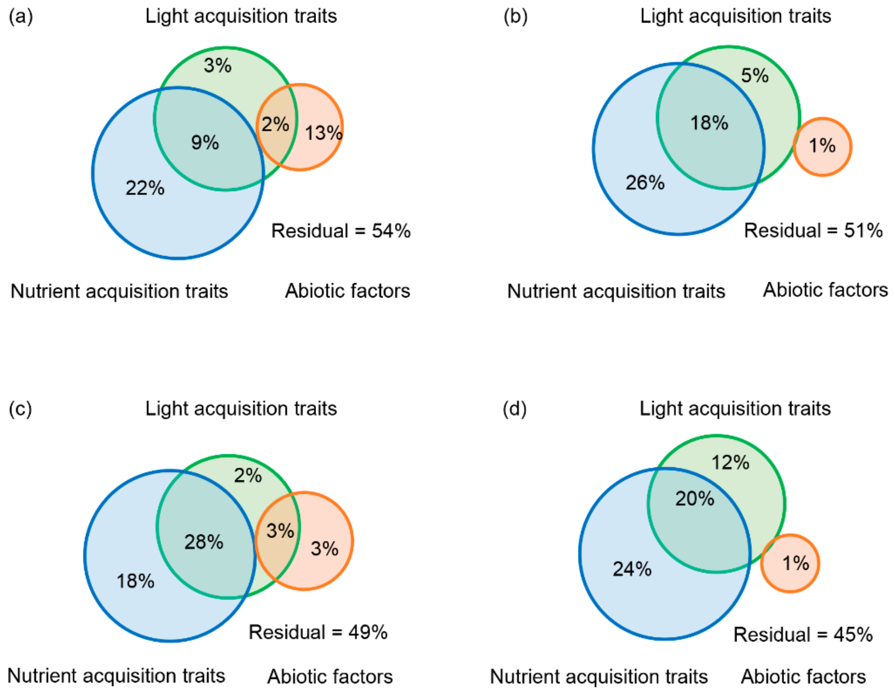

Figure S6 Original structural equation modellings of N addition and plant species richness on first flowering day (a), last flowering day (b), flowering duration (c), and flower number (d). Red and black arrows represent significant positive and negative pathways, respectively. Solid and dashed arrows indicate significant and non-significant pathways, respectively. Numbers near the arrow indicate the standardized path coefficients indicating the effect size of the relationship with * indicating $P <$ 0.05, ** indicating $P < 0.01$, and *** indicating $P < 0.001$. Arrow width is proportional to the strength of the relationship. R^2 represent the proportion of variance explained for each dependent variable. The goodness-of-fit statistics for the structural equation modelling are shown below each model. N represents nitrogen addition.

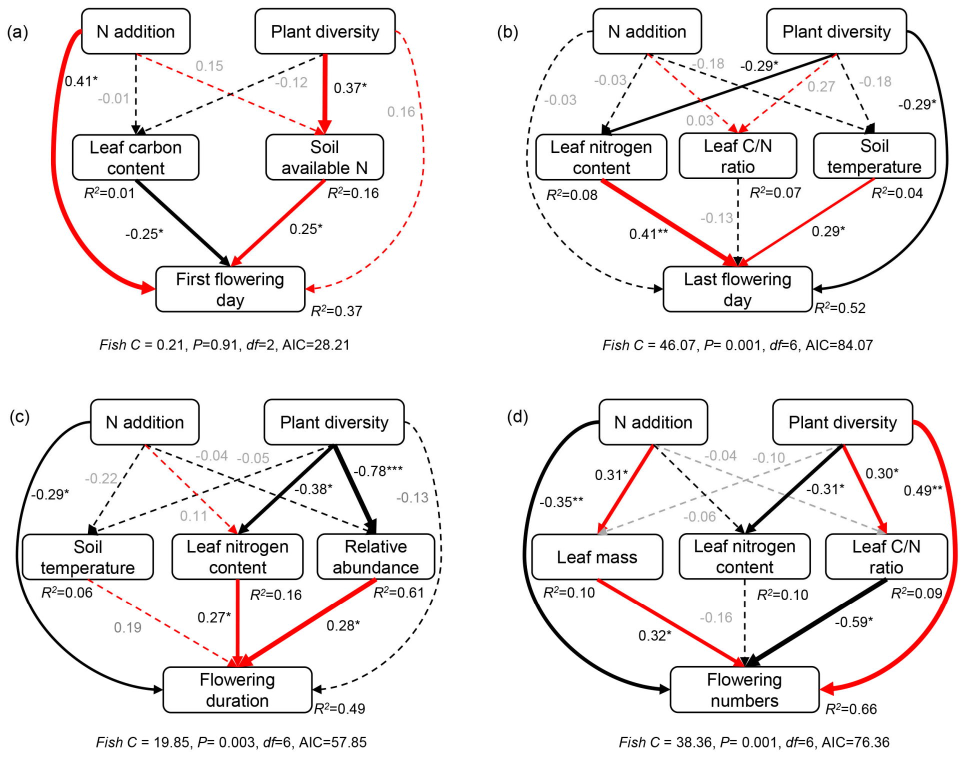

Table S1 Assemblage types at different species richness levels.

Species	Germination rate	SR (1)				SR (2)				SR (4)				SR (6)				SR (8)
		**1**	**2**	**3**	**4**	**1**	**2**	**3**	**4**	**1**	**2**	**3**	**4**	**1**	**2**	**3**	**4**	**1**
Poa annua	73.3%	V				V	V			V	V			V	V			V
Carex breviculmis	65.2%		V					V	V			V	V			V	V	V
Medicago sativa	75.8%			V			V	V			V		V		V		V	V
Astragalus adsurgens	76.7%								V	V		V		V		V		V
Chrysanthemum maximum	59.2%				V	V				V	V	V	V	V	V	V	V	V
Penstemon campanulatus	60.0%										V		V	V	V	V	V	V
Dianthus barbatus	87.2%									V		V		V	V	V	V	V
Allium schoenoprasum	51.9%													V	V	V	V	V

Numbers with different colour represent different assemblage types, numbers in the brackets represent species richness. SR, species richness.
